# The Effect of Cannabidiol (CBD) on the Modulation of Proteolytic Activity on the Honey Bee Workers’ Cuticle

**DOI:** 10.3390/molecules31142498

**Published:** 2026-07-17

**Authors:** Patrycja Staniszewska, Adam Staniszewski, Anna Gryboś, Maciej Sylwester Bryś, Aneta Strachecka

**Affiliations:** Department of Invertebrate Ecophysiology and Experimental Biology, Faculty of Environmental Biology, University of Life Sciences in Lublin, 20-950 Lublin, Polandmaciej.brys@up.edu.pl (M.S.B.);

**Keywords:** proteases, protease inhibitors, immunoproteins, body surface, cannabidiol, hemp extract, *Apis mellifera*

## Abstract

The aim of the study was to determine the effect of cannabidiol extract (CBD) on the activity of the proteolytic system and the concentrations of total proteins on the cuticular surface of worker bees. Marked one-day-old workers were introduced into established colonies, which were divided into three groups (two hives each): CSy (CBD in sugar syrup), CSt (CBD-soaked strip), and C (control, pure syrup). Every seven days, the bioactive cuticular layer was washed off from the marked bees for biochemical analysis. Total protein concentrations increased with worker age in C, CSy, and CSt, and were highest from day 7 in group C. Protease activities increased until day 21/28 of worker life in all groups. From day 21 for acidic proteases, day 28 for neutral proteases, and day 14 for alkaline proteases, the activities were highest in CSy. The activities of neutral and alkaline protease inhibitors decreased with bee age and were always higher in CSy and CSt than in C, regardless of worker age. CBD favorably modulates the proteolytic system on the cuticle of honeybees.

## 1. Introduction

Honeybees, as key pollinators, currently face numerous factors that negatively impact their quality of life. Despite the large number of apiaries in many regions, maintaining colonies in good condition, meaning an adequate number of healthy individuals with a biologically appropriate lifespan, has become a challenge [1]. These problems had “cradle” from the declining immunity of these pollinators, as noted by many scientists. The effectiveness of immune processes depends on the ability of bees to adapt to rapidly changing environmental conditions influenced by climate and anthropogenic factors (industrialization, chemicalization). Bees in this state are unable to respond appropriately to emerging natural pathogens and parasites (e.g., *Vairimorpha/Nosema* spp., *Varroa destructor*, *Paenibacillus larvae*, etc.), which limits their defenses and shortens their lifespan [2]. In recent years, the scientific community has focused on solutions that support and stimulate these processes using biostimulants. Natural supplements, designed to be safe not only for the colony but also for its products, are becoming increasingly popular. Often, these substances have already been positively tested in other animal groups and humans. This reduces the risk of future consumption of bee products containing residues from the decomposition of biostimulants with antioxidant properties, such as piperine, curcumin, caffeine, coenzyme Q10, or substances for so-called “superfoods” like resveratrol or even “pickles” [3,4,5,6,7,8]. In our previous work we have shown that an example of such a substance is the hemp-derived CBD [9]. Its popularity and number of potential health-promoting applications are growing year by year. Demand for these characteristics and properties of the preparation are evident in ongoing attempts to optimize the production of these preparations [10,11,12,13]. To date, CBD has been used primarily for regenerative purposes, supporting the treatment of conditions (e.g., cancer), and rheumatological and neurological conditions (Alzheimer’s, multiple sclerosis, anxiety, sleep disorders) [14]. Recent research also demonstrates new potential for CBD. Dehner et al. (2025) reported significant immune modulation capabilities through changes in morphology (a decrease in leukocyte count in both sexes and changes in mean hemoglobin concentration per red blood cell, mean red blood cell volume, and percentage of neutrophils and monocytes) and alanine transaminase activities, depending on sex in the rats [12]. We also confirmed these properties in honeybees after supplementation in apiary conditions [15,16]. Interestingly, the study also noted the sequence of extract accumulation, indicating the highest levels in the brain, then the kidneys and finally the skin [12]. Other studies on cytokine storm-induced hyperinflammation have shown that CBD reduces pro-inflammatory cytokines (in human PBMC), demonstrating a reduction in the pro-inflammatory cytokines TNFα and IL-1β and a concomitant increase in the anti-inflammatory cytokine IL-10 in *in vivo* mouse studies [11].

Research on this extract is mostly conducted on vertebrates, where the presence of CB1/CB2 receptors has been described. In invertebrates, the actual process by which these compounds act on their bodies is not yet well understood. Specific cannabinoid receptors have not yet been observed in insects. Studies conducted on one of the most popular insect model organisms, Drosophila melanogaster, mainly suggested that these flies may have a preference for consuming CBD-laced food, and in other studies, this extract may affect their circadian rhythm and lifespan, and modulate motor functions [17,18]. An interesting example of CBD’s effects in a more detailed context was conducted on another group of invertebrates—*Caenorhabditis elegans*. A 2024 study described that *C. elegans* possesses systems useful for modeling the effects of cannabinoids, including the NPR-19 receptor, considered an ortholog functionally related to CB1, and TRP channels; CBD also reduced the response to oxidative stress induced by a high-fat diet [19]. However, there is still no clear information regarding the specific classical receptors, as in the case of vertebrates. Nevertheless, it is worth emphasizing that some patterns in studies on vertebrates and invertebrates published by other scientists, as well as our previous studies on CBD extract, have similar conclusions regarding the effects of its use.

As with immune processes in vertebrates, many patterns and processes are reflected in the body of honeybees. One of the first immune mechanisms in bees is the proteolytic system. The proteolytic system consists of proteolytic enzymes (e.g., serine protease, aspartate protease, metalloproteases) and protease inhibitors. Proteolytic enzymes are designed to inhibit and degrade proteins of pathogenic organisms by cleaving at specific bond sites in these compounds. Protease inhibitors, on the other hand, protect bee proteins (including the aforementioned immune proteins, e.g., those with antimicrobial activities) by inactivating pathogen proteases. Proteases and inhibitors also play a crucial role in modulating other cellular processes, such as those related to melanization, phagocyte activation, and lysozyme and peroxidase activities [20,21,22,23]. Although these systems and the synthesis of their components take place in the fat body of bees and are then distributed through the hemolymph, proteolytic activities have also been observed on the insect’s body surface, on the cuticle, where it constitutes the first bioactive immune barrier, supporting physiological and anatomical barriers [22,24,25]. This first-line defense structure is supported internally by supplements such as CBD extract. In previously published studies, we demonstrated the positive effect of the extract on the proteolytic system and other systems, such as the antioxidant system, as well as an increase in concentrations of total proteins in bee hemolymph under cage experiment conditions (*in vitro*) and in apiary conditions (*in vivo*) [9,16]. The proteolytic enzymes and proteins produced in the fat body are transported outside the bee’s body through glands located between the tergites. In this way, these enzymes create an immune layer on the outside of the body. Effective synthesis of proteolytic system compounds is not always synonymous with their high activities and action, also on the cuticle. Effective synthesis of proteolytic compounds does not always equate to their high activities and action, including on the cuticle. Hence, biostimulants are sought that will support these mechanisms. Since anatomical and physiological barriers (i.e., the cuticle, digestive system, and respiratory system) constitute a “defense wall” in the bee’s body against pathogen penetration, they should be strengthened. We hypothesize that since CBD increases the activities of proteolytic compounds in the bee’s hemolymph, it may have a similar effect as part of the protective cuticle biofilm [26]. Therefore, the aim of our study was to determine the activity of the proteolytic system and the concentrations of total proteins in the cuticle of honeybee workers, as the outermost protective barrier shaped by external and internal processes.

## 2. Results

**Figure 1:**
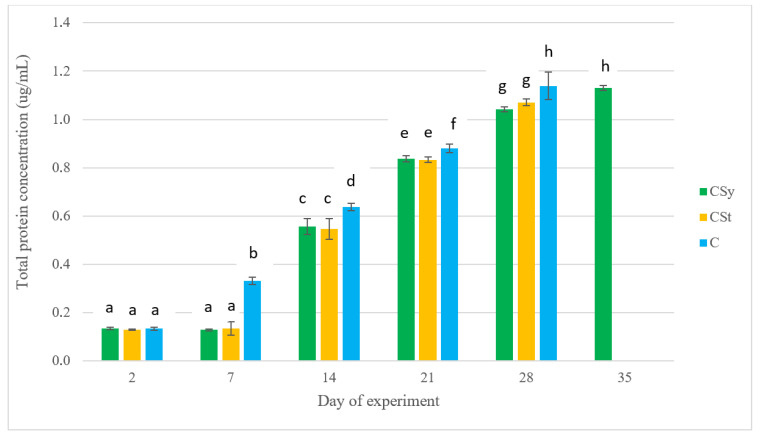
Protein concentrations in the 2-, 14-, 21-, 28- and 35-day-old workers’ protein surface after two methods of supplementation with CBD extract: C—control (pure sugar syrup), CSy—CBD extract in syrup, CSt—CBD extract on strips (two-way ANOVA): day of experiment: F_(4,141)_ = 10,013, *p* = 0.000, se ± 0.003990; supplementation method*days of experiment F_(8,141)_ = 38.956, *p* = 0.0000), se ± 0.006910; one-way ANOVA: supplementation method: F_(2,154)_ = 0.84791, *p* = 0.43030, se ± 0.049263–0.055660. Small letters above the graph bars indicate statistically significant differences between all values obtained during the experiment.

**Figure 2:**
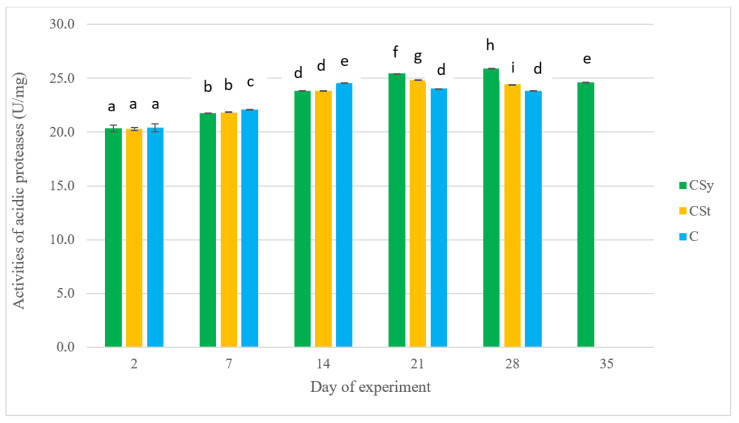
Activities of acidic proteases in the 2-, 14-, 21-, 28- and 35-day-old workers’ protein surface after two methods of supplementation with CBD extract: C—control (pure sugar syrup), CSy—CBD extract in syrup, CSt—CBD extract on strips (two-way ANOVA): day of experiment: F_(4,141)_ = 7272.0, *p* = 0.0000, se ± 0.024339–0.039433; supplementation method*days of experiment F_(8,141)_ = 229.96, *p* = 0.0000), se ± 0.039433–0.047132; one-way ANOVA: supplementation method: F_(2,154)_ = 2.5759, *p* = 0.07936, se ± 0.233372–0.263679. Small letters above the graph bars indicate statistically significant differences between all values obtained during the experiment.

**Figure 3:**
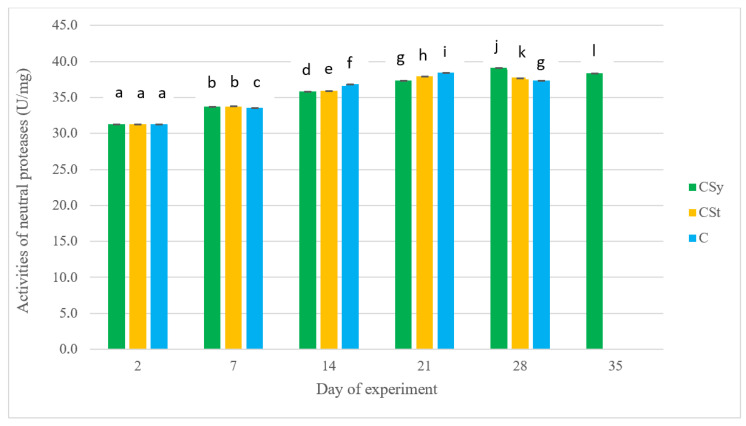
Activities of neutral proteases in the 2-, 14-, 21-, 28- and 35-day-old workers’ protein surface after two methods of supplementation with CBD extract: C—control (pure sugar syrup), CSy—CBD extract in syrup, CSt—CBD extract on strips (two-way ANOVA): day of experiment: F_(4,141)_ = 2070 × 10^3^, *p* = 0.0000, se ± 0.002005–0.003474; supplementation method*days of experiment F_(8,141)_ = 27,974, *p* = 0.0000, se ± 0.003474–0.004152; one-way ANOVA: supplementation method: F_(2,154)_ = 0.91313, *p* = 0.40343, se ± 0.347004–0.380124. Small letters above the graph bars indicate statistically significant differences between all values obtained during the experiment.

**Figure 4:**
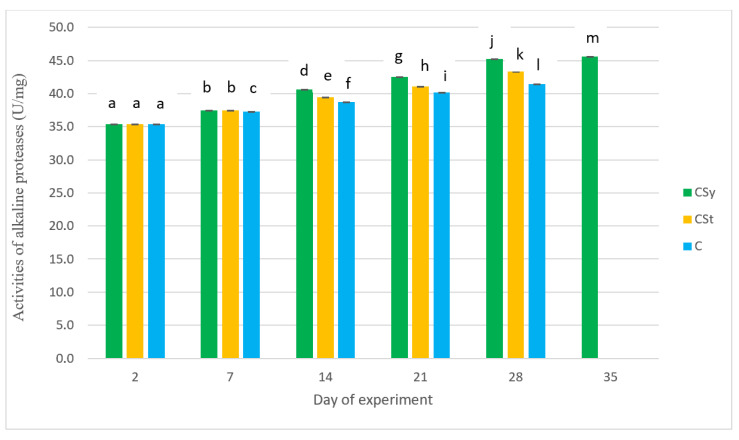
Activities of alkaline proteases in the 2-, 14-, 21-, 28- and 35-day-old workers’ protein surface after two methods of supplementation with CBD extract: C—control (pure sugar syrup), CSy—CBD extract in syrup, CSt—CBD extract on strips (two-way ANOVA): day of experiment: F_(4,141)_ = 2210 × 10^3^, *p* = 0.0000, se ± 0.002197–0.003560; supplementation method*days of experiment F_(8,141)_ = 46,811, *p* = 0.0000, se ± 0.003560–0.004255; one-way ANOVA: supplementation method: F_(2,154)_ = 11.172, *p* = 0.00003, se ± 0.395807–0.447209. Small letters above the graph bars indicate statistically significant differences between all values obtained during the experiment.

**Figure 5:**
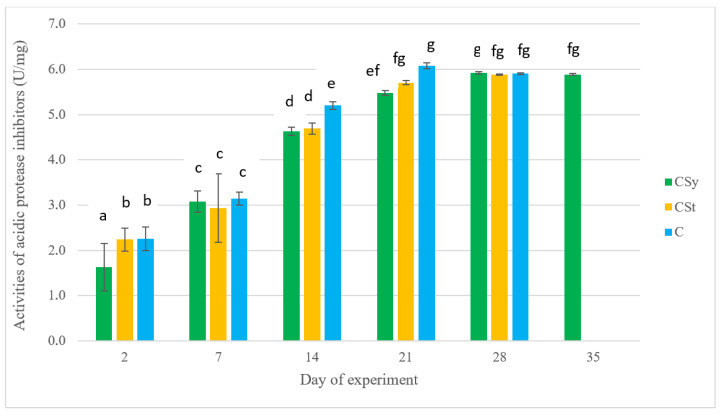
Activities of acidic protease inhibitors in the 2-, 14-, 21-, 28- and 35-day-old workers’ protein surface after two methods of supplementation with CBD extract: C—control (pure sugar syrup), CSy—CBD extract in syrup, CSt—CBD extract on strips (two-way ANOVA): day of experiment: F_(4,141)_ = 1213.4, *p* = 0.0000, se ± 0.048278–0.083620; supplementation method*days of experiment F_(8,141)_ = 5.3497, *p* = 0.00001, se ± 0.083620–0.099945; one-way ANOVA: supplementation method: F_(2,154)_ = 0.13894, *p* = 0.87039, se ± 0.203082–0.229455. Small letters above the graph bars indicate statistically significant differences between all values obtained during the experiment.

**Figure 6:**
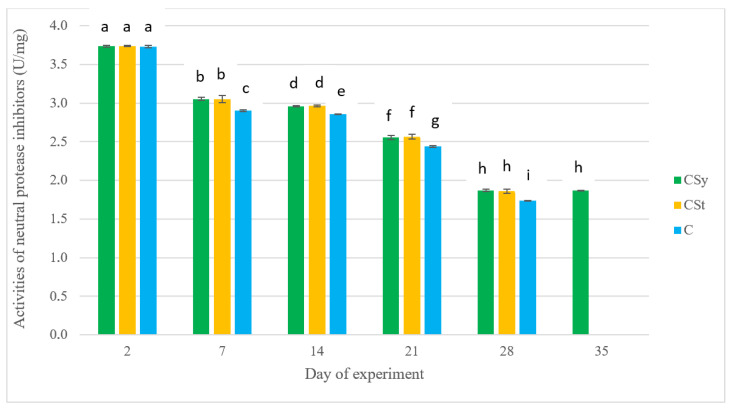
Activities of neutral protease inhibitors in the 2-, 14-, 21-, 28- and 35-day-old workers’ protein surface after two methods of supplementation with CBD extract: C—control (pure sugar syrup), CSy—CBD extract in syrup, CSt—CBD extract on strips (two-way ANOVA): day of experiment: F_(4,141)_ = 36,604, *p* = 0.0000, se ± 0.003550–0.006148; supplementation method*days of experiment F_(8,141)_ = 28.520, *p* = 0.0000, se ± 0.006148–0.007348; one-way ANOVA: supplementation method: F_(2,154)_ = 0.95282, *p* = 0.38792, se ± 0.083056–0.093842. Small letters above the graph bars indicate statistically significant differences between all values obtained during the experiment.

**Figure 7:**
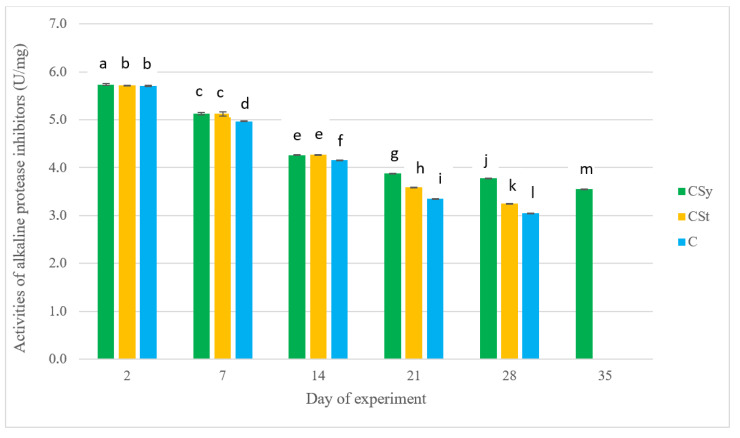
Activities of alkaline protease inhibitors in the 2-, 14-, 21-, 28- and 35-day-old workers’ protein surface after two methods of supplementation with CBD extract: C—control (pure sugar syrup), CSy—CBD extract in syrup, CSt—CBD extract on strips (two-way ANOVA): day of experiment: F_(4,141)_ = 1184 × 10^2^, *p* = 0.0000, se ± 0.002829–0.004900; supplementation method*days of experiment F_(8,141)_ = 1009.7, *p* = 0.0000, se ± 0.004900–0.005857; one-way ANOVA: supplementation method: F_(2,154)_ = 0.09311, *p* = 0.91114, se ± 0.115794–0.130831. Small letters above the graph bars indicate statistically significant differences between all values obtained during the experiment.

For most biochemical parameters measured in this study (except for acid protease inhibitor activities), no significant differences among groups were observed on day 2 of the experiment (Figure 1, Figure 2, Figure 3, Figure 4, Figure 5, Figure 6 and Figure 7).

Total protein concentrations increased with worker age but differed between the supplemented and control groups. No significant statistical differences were observed between the CSy and CSt groups on all dates of analysis. The highest values were achieved by bees that had not been exposed to CBD on day 28 (Figure 1).

Protease activity increased with worker bee age for all proteases until day 21 (Figure 2, Figure 3 and Figure 4). After day 28, activity trends varied among proteases. For all proteases, no significant differences were observed on days 2 and 7 between the supplemented groups, but they were statistically significantly different from the control group (Figure 2, Figure 3 and Figure 4). For acid proteases, activities were higher for the control group until day 14, after which the highest activities were observed for the CSy supplementation (the CSt group had higher values than the control but significantly lower than the CSy group) (Figure 2). Neutral protease activities were highest for the control until day 21, lower for CSt, and lowest for CSy. On day 28, activities of neutral proteases in the control and CSt began to decline, while for CSy it was higher than on day 21 (Figure 3). This trend for CSy was also noted on day 35. The activities of alkaline proteases from day 14 to day 35 was the highest for the CSy group, lower for the CSt group and the lowest for the control group (Figure 4).

In the case of protease inhibitor activity, a decrease in activity with age was noted for neutral and alkaline protease inhibitors, while an increase was observed for acid protease inhibitors (Figure 5, Figure 6 and Figure 7). The activities of acid protease inhibitors increased between days 14 and 21 and were highest for the control group compared to the supplemented groups (the CSt group had higher activity than the CSy group). After day 28, activities of acidic protease inhibitors were the same for all groups until the end of the experiment (Figure 5). The neutral inhibitor activities after day 7 were always the lowest for the control group (in the CSt and CSy groups—no significant statistical differences) (Figure 6). A similar trend was observed for alkaline protease inhibitor activities (the lowest activities for the control group compared to the CSy and CSt experimental groups). From day 21, the activities of these protease inhibitors showed significant differences between the CSy and CSt groups, with the CSy group achieving higher activities than the CSt and control (Figure 7). 

## 3. Discussion

The tests used pure commercial CBD oil, which, in this study and previous studies conducted by our team, demonstrated immune-boosting effects visible in the activities of honeybees’ basic defense systems. The experiment was primarily aimed at moving the study from *in vitro* to *in vivo* conditions, where we could observe and study an experiment based on a design similar to a cage experiment, but with the influence of environmental factors similar to those prevailing among bees living naturally in an apiary, performing all the natural tasks affecting their bodies. As it turned out, comparing the studies conducted *in vitro* and *in vivo*, we obtained promising replication of trends and tendencies between them. Interestingly, the very low standard deviation between the results obtained from individual bees is worth noting, which increases the reliability of the observed activity levels and concentrations. Despite the existence of a study on a cage experiment conducted with hemp extract, the results obtained in an apiary environment experiment with the same design in 2022 [16] are considered a better reference point for comparison.

Two supplementation methods were used in the experiment to test the effectiveness and reproducibility of results, and to approach the practical application of supplementation in beekeeping (the two most popular methods: administration in syrup and on a cloth strip). Supplementation using a CBD-soaked strip showed mostly intermediate values between the control and syrup supplementation. We hypothesize that these intermediate values are due to the time delay in which the extract could develop its effect. Supplementation in syrup allows the active substance to quickly and directly enter the body, where it can begin to act through the intestine, fat body, and hemolymph. In the case of the strips, the substance could have entered the bees’ bodies later at a lower concentration (evaporation of the substance from the strip, or introduction of the substance only as a result of, for example, cleaning the body after rubbing against the substance on the strip). This could have created the possibility of a similar effect, but with an observed “delay in time” compared to syrup. We obtained similar results in other studies of this supplementation in our publications.

In this paper, we present complementary results previously obtained after the use of CBD in bee diets. We believe that this research is important because there is a lack of studies examining the effect of supplementation on the activity of insect cuticular systems, and the activity of the proteolytic system on the insect body surface. Therefore, this work will aid in future research and comparisons of the trends observed between the most important internal and external tissues/immune barriers (i.e., fat body, hemolymph, cuticle).

The results obtained for the body surface activity of the proteolytic system are consistent with the main upward trends in the groups using the supplement compared to the control groups in the study by Skowronek et al. (2022) [16]. However, a visible difference between the studies was observed in the concentration of total protein. Its concentration on the cuticle is lower in the supplemented groups compared to the control group. This trend contrasts with total protein concentration in the hemolymph, where these values increased with the consumption of CBD oil. The high total protein levels in the hemolymph after CBD application, but lower levels in the cuticle, suggest that the biostimulant’s action is sufficient to stimulate internal processes, but the effect may be limited to the synthesis of immune proteins within the body, without the need for external exposure. The concentration of total protein in the bee cuticle is naturally lower than in the hemolymph primarily due to their differing biological roles and composition. Hemolymph, the circulatory fluid of bees, contains a wide variety of proteins involved in transport, immune response, and metabolism, resulting in a higher overall protein concentration. It serves as a medium for distributing nutrients, immune proteins like lectins and enzymes, and other bioactive molecules throughout the bee’s body, which demands a rich and abundant protein content [27,28,29]. In contrast, the cuticle serves as an external protective layer composed mainly of chitin and structural proteins. Its primary function is to provide a physical barrier against environmental threats and prevent water loss. The structural proteins in the cuticle are fewer and more specialized compared to the diverse and abundant proteins in the hemolymph. Additionally, proteins on the cuticle may be more prone to degradation and are present in lower concentrations because the cuticle is not involved in metabolic or immune functions to the same extent as hemolymph [30,31]. Studies show that hemolymph protein concentration varies among species and is linked to physiological functions like immunity and lifespan, highlighting its richness in proteins such as lectins and proteases with antimicrobial and antioxidant activities. The cuticle, being a mostly structural element, naturally has less total protein concentration [28,29,32]. An additional aspect is the aforementioned antimicrobial effect of CBD oil [33]. It is worth noting that supplementation on the strip (CSt), by evaporating and remaining on the bees’ carapace, could have created an additional external biocidal barrier against pathogens, which would have reduced the need for increased protein levels on the body surface (total protein levels on days 14 and 21 were the lowest for the CSt group). The oil used, thanks to its physical characteristics, may also contribute to better adhesion and the formation of a protective layer for lipophilic compounds (sealing effect). The bee cuticle is largely composed of lipids, i.e., waxes, which allowed the tested substance to not only exert its surface action but also to mix and transfer compounds into the natural lipid layer [34,35,36]. It is also worth noting that the results should be considered holistically, considering that high protein concentrations do not always correlate with their activities and positive effects on the body surface. In a 2012 study by Strachecka et al., a year after examining the effects of amitraz and oxalic acid on the active layer of the cuticle, it was found that the negative impact on the organism could have resulted in structural damage, resulting in a higher number of inactive proteins appearing on the cuticle [37]. Similar conclusions were reached in a study using formic acid, where, despite the overall negative impact on the bees’ immune system, higher concentrations of both hydrophilic and hydrophobic cuticle proteins were observed (Strachecka et al., 2015) [3]. From the other hand, CBD also stimulates the proteolytic system (shown in this study), increasing the activity of proteases and their inhibitors, which can lead to more protein degradation on the cuticle surface. This means that although the bee produces more proteins internally, protein levels on the cuticle can decrease due to heightened proteolytic activity breaking down proteins on the surface. Proteases are enzymes that hydrolyze peptide bonds in proteins, effectively degrading them into smaller peptides and amino acids. When protease activity increases, more proteins on the cuticle may be digested and removed, leading to a decrease in overall protein concentration. This mechanism is common in biological systems where proteases are used to degrade extracellular proteins for remodeling, defense, or nutrient recycling [38,39,40,41,42]. In the case of the cuticle, heightened protease activity—such as that stimulated by CBD supplementation—accelerates the breakdown of cuticle surface proteins, thereby lowering their measured levels. 

In the case of protease activities, we observed increased activities of acidic proteases (on day 21), neutral (on day 28), and alkaline proteases (on day 14) for the CSy and CSt groups. In our opinion, there is no such noticeable shift in effects in the CSy group compared to the CSt group. The closeness of the mean values between the activity results suggests that the supplement has a stabilizing effect with a slight stimulation of proteolytic activity on the cuticle. This stabilizing effect and slightly higher activity may allow the bees to experience milder effects of aging. CBD oil may therefore confirm its effect on reducing immunosenescence (aging of the immune system), which may have led to the higher number of individuals in the CSy group (a single measurement on day 35). This thesis is also supported by the high levels of Ca+ ions in the hemolymph of worker bees observed in previous studies in the supplemented groups. Calcium ions, by regulating lipid metabolism, also affect the development and aging of the body (diapause, metamorphosis) [16,43]. It is worth noting that these effects are most pronounced from days 14 to 28. This is a crucial period in the bee’s life, when it prepares for a complete change in function, as it becomes a forager on day 21. Higher protease activity can help the bee combat the negative environmental factors it encounters for the first time [44,45]. It is during this period that the bee requires the greatest immunological activity. Generally speaking, the lipophilic nature of this compound allows it to freely enter the body and act effectively in the fat body, where proteases and inhibitors are also synthesized. These results, consistent with the work of Skowronek et al. (2022) [16], are also consistent with the results obtained for the hemolymph proteolytic system in publications using a supplement with similar physicochemical properties, e.g., coenzyme Q10, but also for other supplements, e.g., caffeine, curcumin [3,4,46].

Protease inhibitor in most CBD groups showed higher mean activity than the control group. The exception was acid protease inhibitors, where the control group’s parameters were exceeded on days 14 and 21. Compared to other protease inhibitors, it appears that contact with the pathogen that triggered the reaction may have occurred during this time. The response of acid protease inhibitors suggests the presence of fungi or viruses against which they must have shown increased activity [44]. Interestingly, the remaining inhibitors show a completely different trend in activity on the cuticle compared to acid inhibitors. The pattern for neutral and alkaline inhibitors suggests their stable decline with age, as if their activity were only preventive in the event of a threat. This trend and average values apply to all groups in the experiment, confirming the actual occurrence of this tendency for the studied colonies. In this case, protease inhibitors may act similarly to proteases, protecting the immune system and stabilizing it in the second half of the bees’ lives (significant differences in activity occur between days 14 and 21 of life). After day 28, we observe stabilization of the parameters compared to day 35 for the CSy group. The stabilization of all parameters may result from the previously mentioned ability to seal the cuticle. Honeybees harden their cuticle with age, but in the second half of life, with reduced glandular efficiency, the production of compounds that form protective barriers also decreases [25,47]. Furthermore, atmospheric conditions also have the effect of “washing out” this layer. The stabilization of the proteolytic system’s activity parameters results not only from the internal synthesis of proteases and inhibitors, but also from the potential additional lipid coating of the cuticle with a protective layer of CBD oil, allowing the bee to maintain the active layer on its body surface for longer. The results of the proteolytic system activity obtained in this manuscript should be compared and assessed comprehensively with the previously mentioned effects obtained after the use of amitraz and formic acid, commonly used in the treatment of the *Varroa destructor* parasite in honeybees. In the case of the first two compounds, they led to the complete disappearance or significant reduction in the activity of natural protease inhibitors—acidic, neutral, and alkaline, including hydrophilic and hydrophobic forms. The authors suggest these changes negatively impact the structure of the protease–substrate–inhibitor complex [37,48].

## 4. Materials and Methods

The experiment was conducted during the summer season. Worker and queen bees were obtained from colonies exhibiting similar biological and breeding parameters, including comparable strength and age (i.e., brood count, queen age, bee population size, frame construction type, and food reserves), as well as no symptoms of infestation with *Varroa destructor* or pathogens of the Vairimorpha/Nosema genus. These colonies had not been subjected to prophylactic treatment against *V. destructor*, allowing the development of natural immune mechanisms without the intervention of pharmacological agents. No *Vairimorpha/Nosema* spp. spores were detected in the samples (detection was performed using a light microscope and prepared microbiological preparations derived from bees).

### 4.1. Apiary Part

#### 4.1.1. Obtaining Queens and Workers for the Experiment

For the experiment, nine mated queen bees were prepared, six of which were used to create test colonies, and three were used to obtain one-day-old worker bees. All queens belonged to the same genetic line—sisters descended from a single source queen. The queen collection procedure was performed according to the methodology described by Skowronek et al. (2023) [9].

Three sister queens were placed in isolation cages with empty combs for 12 h to stimulate egg laying. After egg laying, the queens were returned to their colonies. The combs containing eggs were marked and left in the parent colonies until brood capping. After 20 days, the combs were transferred to an incubator maintained at 35 °C, where one-day-old worker bees hatched. One-day-old bees in the next step were marked with oil markers in three different colors (a given color corresponded to the appropriate experimental/control groups). A detailed description of the procedure can be found in Skowronek et al. (2022) [16].

#### 4.1.2. Colony Preparation for the Experiment

Four-frame wedding hives were used for the experiment, conducted in apiary environment conditions. Fragments of combs were taken from the source colony containing larvae at various stages of development and then matched to the frames in the experimental hives. Additionally, worker bees of various ages (imago) were obtained from the same colony, and 200 individuals were introduced to each hive. This ensured that each study unit contained all developmental stages. One of six previously prepared queens was introduced to each hive. After one month, their egg-laying capacity was assessed, and then marked one-day-old workers from the three remaining sister queens were added to each hive.

#### 4.1.3. Experimental Design

The mating hives were divided into three groups of two mini colonies each.

Previously marked one-day-old worker bees were introduced into six previously prepared test hives. The hives were divided into three experimental groups (two hives per group):

(1) Group CSy—colonies receiving CBD in the form of sugar syrup;
(2) Group CSt—colonies receiving CBD on a cotton carrier;
(3) Control group (C)—colonies fed pure sugar syrup.

#### 4.1.4. Supplement Administration

The experiment used a 30% oily hemp extract containing cannabidiol (CBD), produced by HempOil (CBD content: 3 g per 10 mL of preparation). The extract was obtained using supercritical carbon dioxide extraction (according to the manufacturer’s information). The supplementation regimen for each experimental group was as follows:

(1) CSy: Supplementation occurred ad libitum on days 2, 4, and 6 of the experiment. CBD oil was administered in a mixture with sugar syrup (1:1 sugar–water ratio) and glycerin. The volumetric proportions of the ingredients were 0.01 parts extract:0.5 parts distilled water:0.5 parts glycerin. Preliminary research conducted by the team showed that glycerin added to sugar syrup does not differ from pure sugar syrup.
(2) CSt: A mixture of extract, distilled water, and glycerin was used in a ratio of 0.8:1.5:1.5 (*v*/*v*/*v*). Textile strips (2 × 10 cm in size) were soaked in 10 mL of the mixture and then placed inside the hives. Supplementation occurred on days 2, 4, and 6 of the experiment. A detailed description of the composition of supplements and their preparation is available in the work of Skowronek et al. (2022) [16].

### 4.2. Laboratory Part

#### 4.2.1. Collection of Bees for Biological Material

Ten marked worker bees were collected weekly from each of the six experimental colonies (60 individual bees in total in each week). Samples were collected on days 2, 7, 14, 21, and 28 of the experiment (for groups CSt, CSy, and C) and additionally on day 35 for group CSy. Intact bees (no damage) were frozen for the purpose of obtaining biological material (surface active layer) from the cuticle.

#### 4.2.2. Collection of Surface Active Layer from Bees

Surface protein collection was performed according to the method of Łoś & Strachecka (2018) [49]. Frozen bees were placed in 2 mL Eppendorf tubes and then flooded with 1.5 mL of Triton™ X-100 reagent (Sigma-Aldrich, St. Louis, MO, USA). In the next step, each tube containing a bee was vortexed for 2 min. After this step, the bees were removed from the Eppendorf tubes, and the remaining fluid collected from the bees’ body surface was frozen until biochemical analysis [49].

#### 4.2.3. Biochemical Analyses

The following biochemical analyses were performed for all individual samples:

(1) Total protein concentration was determined using the Lowry method, as modified by Schacterle (Schacterle et al., 1973) [50].
(2) Proteolytic system activity was determined as follows:
  - Activities of acidic, neutral, and alkaline proteases according to the Anson method, modified by Strachecka [49,51];
  - Activities of natural inhibitors of acidic, neutral, and alkaline proteases according to the Lin method.

### 4.3. Statistical Analyses

The results were analyzed using Statistica formulas, version 13.3 (2017) for Windows, StatSoft Inc., Tusla, OK, USA. The one/two-way ANOVA followed by post hoc Tukey HSD tests (*p*  =  0.05) were used to compare the results for each basic immunity system parameter (total protein concentration, protease activities, inhibitor protease activities) of honeybee workers depending on the method of administration to the control group (control—C, strip—CSt and syringe—CSy) and the day (day 2, 7, 14, 21, 28, 35) of supplementation with hemp extract. Normal distribution was checked using the Shapiro–Wilk test and homogeneity of variance was assessed by the Levene test.

## 5. Conclusions

Based on the obtained results, CBD oil used in supplementation of honeybee workers demonstrates a positive modulation of the proteolytic system, also visible in the activity of the cuticular proteolytic system. This positive effect may be due to improved synthesis of proteases and their inhibitors in the hemolymph of these insects after supplementation, as well as the surface sealing and antimicrobial effects of the aforementioned cannabidiol.

Although these studies overall demonstrate a positive effect of CBD on many parameters related to immunity in honeybees, they do not provide a definitive answer regarding the role and detailed process of action of cannabis compounds (action on biochemical pathways, reception and functioning of receptors) in the health of invertebrates, including insects.

## Data Availability

The datasets and materials that have been used, analyzed and presented in this manuscript are not publicly available but available at the University of Life Sciences in Lublin. At a justified request of the interested party, they may be made available by the corresponding author.
